# Optimal Color Design of Psychological Counseling Room by Design of Experiments and Response Surface Methodology

**DOI:** 10.1371/journal.pone.0090646

**Published:** 2014-03-04

**Authors:** Wenjuan Liu, Jianlin Ji, Hua Chen, Chenyu Ye

**Affiliations:** Department of Psychological Medicine, Zhongshan Hospital, Fudan University, Shanghai, China; Rutgers University, United States of America

## Abstract

Color is one of the most powerful aspects of a psychological counseling environment. Little scientific research has been conducted on color design and much of the existing literature is based on observational studies. Using design of experiments and response surface methodology, this paper proposes an optimal color design approach for transforming patients’ perception into color elements. Six indices, pleasant-unpleasant, interesting-uninteresting, exciting-boring, relaxing-distressing, safe-fearful, and active-inactive, were used to assess patients’ impression. A total of 75 patients participated, including 42 for Experiment 1 and 33 for Experiment 2. 27 representative color samples were designed in Experiment 1, and the color sample (L = 75, a = 0, b = -60) was the most preferred one. In Experiment 2, this color sample was set as the ‘central point’, and three color attributes were optimized to maximize the patients’ satisfaction. The experimental results show that the proposed method can get the optimal solution for color design of a counseling room.

## Introduction

Color, one of the most powerful aspects of the environment, has been reported to promote human adaptation to the environment and enhance spatial form [1]. Color may change the perceived size and warmth of a room, elicit associations, enhance introversion or extroversion, incite anger of relaxation, and influence physiological responses [2], [3]. Several studies have been conducted to study the psychological impact of color. For example, whereas warm colors provide visual activation and stimulation, cool colors communicate subtlety and relaxation.

The visual environment is a vital element influencing hospital staff morale and productivity; studies have even reported that an enhanced visual environment have produced improved faster recovery rates by as much as 10%. In fact, these improvements have been attributed to particular elements of the visual environment; they include the use of appropriate color in interior design. In hospital design, color can have an impact on peoples’ perceptions and responses to the environment and also affect patient recovery rates, improving the quality and overall experience of patients, staff and visitors. Color is also powerful tools for coding, navigation and wayfinding, color can also promote a sense of well-being and independence.

There are numerous studies in the field of psychology which demonstrate the relationship between human behavior and color. However, color studies in the environmental design field are almost non-existent. Nourse and Welch [4] tested Wilson’s [5] finding that red is more arousing than green. Jacobs and Hustmyer [6] found red was significantly more arousing than blue or yellow, and green more than blue. Fehrman and Fehrman [7] reported on the effects of blue, red, and yellow on task performance and arousal for the effective color selection of interior spaces. Kwallek, Lewis, and Robbins [8] examined the effects of a red colored office versus a blue one on subject productivity and mood. Kwallek and Lewis [9] investigated effects of environmental color on gender using a red, white, and green office. The experiment assessed worker performance in proofreading and mood under different colored office environments. Weller and Livingston [10] examined the effect of colored-paper on emotional responses obtained from questionnaires. Six different questionnaires were designed and compiled in this order: pink-guilty, pink-not guilty; blue-guilty, blue-not guilty; white-guilty, white-not guilty. Boyatzis and Varghese [11] investigated children’s color and emotion associations. They found that children showed positive emotions to bright colors (pink, red, yellow, green, purple, or blue) and negative emotions for dark colors (brown, black, gray). Hemphill [12] examined adults’ color and emotion associations and compared them with the findings by Boyatzis and Varghese [11].

Scientific research means reviewing a concept through observation in order to test a theory or hypothesis in order to explain a phenomenon. The presupposed theory and hypotheses are tested by systematic observations to make a general explanation for the natural phenomena. Experiments are useful to explore the phenomena because they involve testing hypotheses and investigating cause-and-effect relationships [13]. Experiments are characterized by the manipulation of independent variables and identifying possible cause-and-effect relationships between an independent variable and a dependent variable. Types of experiments are categorized by the degree of random assignment of subjects to the various conditions; they are true experiments, quasi-experiments, and single-subject experiments. True experiments require unbiased random assignment of subjects to treatment groups and the researcher’s ability to manipulate independent variables directly. Rigorous experiments are typically done in a laboratory where it is possible to control variables. The major advantage of these experiments is their ability to establish causal relationships; quasi-experiments do not establish causal relationships to the same degree as true experiments since experiments in the field routinely encounter uncontrollable factors. The advantage of quasi-experiments is their higher generalizeability because of their naturalness when compared to the artificiality of true experiments [14].

Based on previous studies, the consistent trend is that blue and green are the most preferred colors. However, the majority of color preference studies failed to control the confounding variables such as color attributes [15], [16]. A well-controlled color preference study of psychological patients appears to be non-existent. In order to address the limitations found in previous research, this study is the first to use experimental design in order to provide a foundation for color studies. This research advances the understanding of the value of color in a counseling room by studying psychological patients’ perceptions of color. This knowledge should facilitate improvements in the design of hospitals. The main purposes of this study are: 1) to propose an experiment-based color design research approach, 2) to create an optimization solution on the use of color design.

## Methods and Materials

### Ethics statement

All studies were approved by Institutional Review Board (IRB) at the Zhongshan Hospital, and all participants provided written informed consent.

### Conceptual Model of the Study

Sherman, Shepley, and Varni [17] proposed a conceptual model which indicated that physical environments can impact patients’ outcome. They proposed that this relationship between the physical environment and patients’ outcome is mediated by environmental satisfaction. The role of environmental satisfaction as a mediator between the physical environment and patients’ outcome is considered a significant indicator and this conceptualization is well supported by Thurber and Malinowski [18], Whitehouse et al [19], and Boman and Enmarker [20].

**Figure 1:**
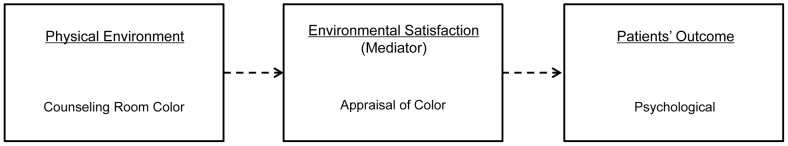
Conceptual model of the impact of color on patients’ outcome.

Drawing on the model presented by Sherman, Shepley, and Varni [17], the impact of color on patients’ outcome can be explained as seen in Figure 1. Figure 1 describes that color preference can mediate patients’ outcome through their satisfaction with colors.

### Overview of experiments

A total of 75 patients with normal or corrected-to-normal vision participated, including 42 for Experiment 1 and 33 for Experiment 2.

The color samples were rendered using the software Photoshop on a Core i3 computer (HP ProBook 6450b) equipped with an AMD Radeon HD 6370 graphics adaptor. It was displayed on a large rear projection screen (2.44 m horizontally, 1.85 m vertically). The projection allowed for stereoscopic viewing by use of two projectors with a resolution of 1,920×1,080 pixels each and a color depth of 32 bits. The refresh rate of the display was 60 Hz for each eye. The participant was seated at a distance of 2.0 m from the projection screen.

Design Expert software was utilized to conduct the experimental design and analysis. The software is performed on a Pentium 4 PC with a 2.4 GHz CPU and 512 MB RAM.

The color system of Commission International de l’Eclairage (CIE) is chosen because its color space is more symmetrical than other color systems, such as Munsell color system and Ostwald color system [21], [22].

In 1976, CIE recommended two standard color difference formulae: CIELAB and CIELUV for the colorant and lighting industries, respectively. Each of these two formulae also provides a uniform color space for representing colors in a perceptually even fashion.

**Figure 2:**
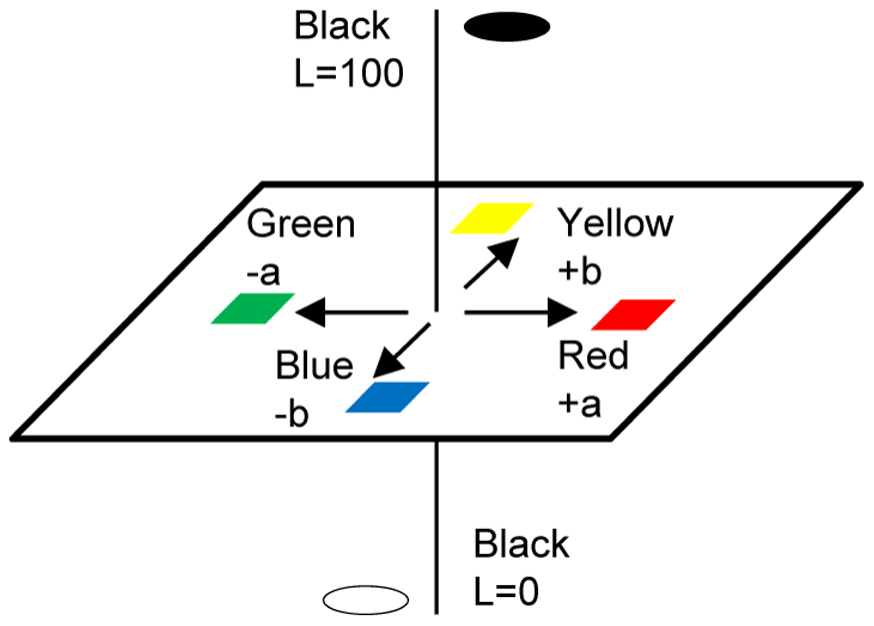
CIE Lab color space.

The CIELAB space has been up to now the most widely used for presenting colors. Figure 2 shows a three dimensional CIELAB space. The neutral scale is located in the center of the color space. The L values of 0 and 100 represent a black and a reference white, respectively. The a and b values represent the redness–greenness and yellowness–blueness attributes, respectively.

**Figure 3:**
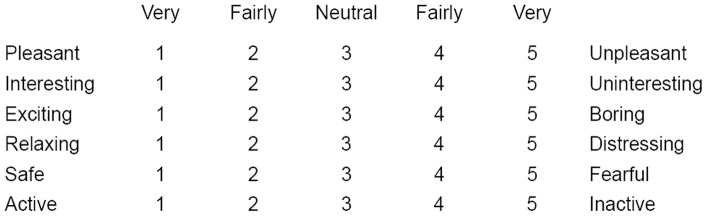
The questionnaire of Affective Appraisal Scale.

We employed the Affective Appraisal Scale [23], [24] to investigate the differences of participants’ impressions of the environment across the four conditions and to check the manipulation of the interior physical appearance as independent variables. The scale had six 5-point bipolar adjectives to assess the affective appraisals of the environment: pleasant–unpleasant, interesting– uninteresting, exciting–boring, relaxing–distressing, safe–fearful, and active– inactive. 1 and 5 stood for very, 2 and 4 for fairly, and 3 for neutral. The ratings were recorded by respondents on a specially prepared questionnaire (as shown in Figure 3). Participants were told that since there are no right or wrong answers, they should give their honest opinions.

### Experiment 1

**Figure 4:**
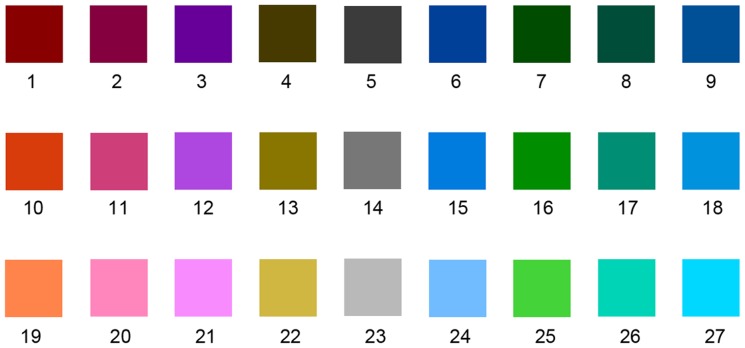
Experiment 1: Color samples.

With 3 attributes of color, 3 values of Lightness (L = 25, 50, and 75; 0≤L≤100), 3 values of a (a = –60, 0, and 60; –120≤a≤120), and 3 values of b (b = –60, 0, and 60; –120≤b≤120) are selected to generate 27 ( = 3×3×3) representative color samples (as shown in Table 1) in this study. Figure 4 shows these color samples.

**Table 1:** Experiment 1: Results of color sample selection.

ID	*L*	*a*	*b*	pleasant–unpleasant	interesting–uninteresting	exciting–boring	relaxing–distressing	safe–fearful	active–inactive	Avg. Score
	25	60	60	4.5	4.6	4.1	4.3	3.7	3.4	4.1
2	25	60	0	4.3	4.2	4.1	4.2	3.5	3.7	4.0
3	25	60	–60	4.2	4.4	4.3	4.7	4.1	4.0	4.3
4	25	0	60	4.9	4.7	4.9	4.0	4.0	4.7	4.5
5	25	0	0	4.9	4.8	4.9	4.2	3.5	4.8	4.5
6	25	0	–60	4.4	3.5	4.3	4.5	4.1	4.3	4.2
7	25	–60	60	4.4	4.0	4.4	4.6	3.5	4.3	4.2
8	25	–60	0	4.3	4.3	4.7	4.4	3.2	4.5	4.2
9	25	–60	–60	3.9	3.2	4.5	4.3	3.7	4.1	4.0
10	50	60	60	2.5	2.6	2.0	4.1	4.2	3.0	3.1
11	50	60	0	2.5	2.4	2.1	4.2	4.1	2.9	3.0
12	50	60	–60	2.7	2.6	3.0	3.9	4.0	3.2	3.2
13	50	0	60	2.9	3.5	3.9	3.1	3.2	3.9	3.4
14	50	0	0	3.5	4.0	4.3	3.9	3.0	4.4	3.9
15	50	0	–60	2.3	2.1	3.2	3.0	2.7	3.4	2.8
16	50	–60	60	3.3	2.8	3.7	3.0	2.7	3.6	3.2
17	50	–60	0	3.4	2.7	3.5	2.8	2.5	3.5	3.1
18	50	–60	–60	2.8	2.3	3.0	2.9	2.4	3.0	2.7
19	75	60	60	1.3	1.9	1.9	3.9	3.9	2.5	2.6
20	75	60	0	1.4	1.7	1.8	4.0	3.7	2.3	2.5
21	75	60	–60	1.5	2.0	2.1	4.0	3.8	2.1	2.6
22	75	0	60	2.5	2.4	3.7	2.8	2.3	3.4	2.9
23	75	0	0	2.6	2.9	3.9	2.5	2.1	3.9	3.0
24	75	0	–60	1.7	2.6	1.9	1.7	1.5	2.7	2.0
25	75	–60	60	1.7	2.7	1.9	1.8	2.2	2.3	2.1
26	75	–60	0	1.9	2.4	2.3	1.9	2.2	2.2	2.2
27	75	–60	–60	2.0	2.1	2.1	2.0	2.2	2.2	2.1

### Experiment 2

The next step to improve color parameters was to apply a response surface method (RSM) using a central composite design (CCD), which were referred to NIST/SEMATECH. The work which initially generated interest in the package of techniques was a paper by Box and Wilson [25]. The experimental design used in this study was the central composite design (CCD) to obtain a second-order regression model.

**Figure 5:**
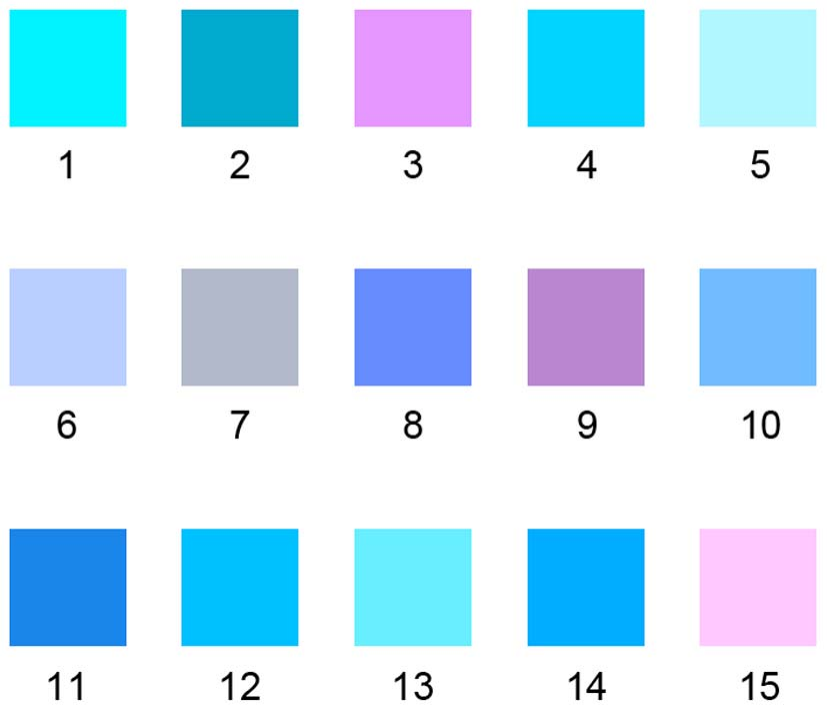
Experiment 2: Color samples.

The design ranges for L, a, and b are [62.5, 87.5], [–30, 30], and [–90, –30] respectively. Design Expert was utilized to conduct the experimental design and analysis. The design, consisting of 15 experimental runs (including one center points, six axis points and eight factorial points), is summarized in Table 2. The color samples are shown in Figure 5.

**Table 2:** Experiment 2: Matrix for the CCD and results.

ID	*L*	*a*	*b*	pleasant–unpleasant	interesting–uninteresting	exciting–boring	relaxing–distressing	safe–fearful	active–inactive
1	88	–30	–90	1.9	2.3	2.2	4.1	2.2	1.2
2	63	–30	–30	2.4	2.2	3.9	2.3	3.1	3.9
3	75	50	–60	4.5	3.6	1.2	4.9	4.7	2.6
4	75	–50	–60	2.9	1.3	2.1	4.3	2.9	2.9
5	96	0	–60	1.2	3.9	3.9	3.0	2.1	2.3
6	88	30	–90	3.3	4.3	4.2	2.5	4.2	2.4
7	75	0	–10	3.0	4.8	4.8	1.4	4.8	4.7
8	63	30	–90	3.8	2.3	2.5	3.1	3.0	3.8
9	63	30	–30	3.8	3.8	2.0	4.7	4.3	3.9
10	75	0	–60	1.9	2.7	1.4	1.3	1.2	2.8
11	54	0	–60	3.1	3.2	3.1	3.1	3.4	3.9
12	75	0	–110	2.0	1.2	2.4	2.7	2.8	2.9
13	88	–30	–30	2.1	3.4	3.7	4.1	1.9	2.2
14	63	–30	–90	2.1	1.8	2.1	2.3	2.8	3.9
15	88	30	–30	2.9	4.1	2.1	2.2	4.2	2.1

RSM is a collection of experimental strategies, mathematical methods, and statistical inference that enables an experimenter to make efficient empirical exploration of the system of interest [26].

Because the functional relationship between the response and inputs is unknown and perhaps complicated, the first step in RSM is usually to start with the first-order designs.

In regions of higher curvature, especially near the optimum, second-order models are commonly used:

To determine the parameters that optimize the multiple responses, the desirability function approach is utilized. The desirability function approach proposed by Derringer and Suich [27] is one of the most widely used methods for the optimization of multiple response processes. In this study, the optimization procedure was to minimize 6 responses simultaneously.

## Results

In Experiment 1, results are listed in Table 1. The best color sample for ‘pleasant-unpleasant’ is option 19. Similarly, the best candidates for other 5 indices are option 20, option 20, option 24, option 24, and option 21 respectively. Although the performance of option 20 and option 24 are good on 2 indices, it is not concluded that the two options are the final best solution for multiple-objective optimization. We should use the RSM-based color parameter optimization procedure to find the truly best candidate. In this paper, we suppose that 6 indices are equally important, and then the option 24 is selected as the basic color sample.

In Experiment 2, analyses of variance for response variables and significant factor estimations using least squares analysis were generated by the Design Expert program. Table 3 summarizes the experimental results for pleasant-unpleasant response. The Model F-value of 7.62 implies the model is significant. There is only a 1.88% chance that a ‘Model F-Value’ this large could occur due to noise. ‘Adeq Precision’ measures the signal-to-noise ratio. A ratio greater than 4 is desirable. The ratio of 9.941 indicates an adequate signal. This model can be used to navigate the design space.

**Table 3:** Model summary statistics.

Source	Sum of squares	df	Mean square	*F* value	Prob>*F*
Model	10.41	9	1.16	7.62	0.0188
Residual	0.76	5	0.15		
Corr. total	11.17	14			
Standard deviation	0.39		*R*-squared	0.9321	
Mean	2.73		Adj. *R*-squared	0.8098	
CV%	14.28		Pred. *R*-squared	0.4211	
Press	6.47		Adeq. precision	9.941	

**Figure 6:**
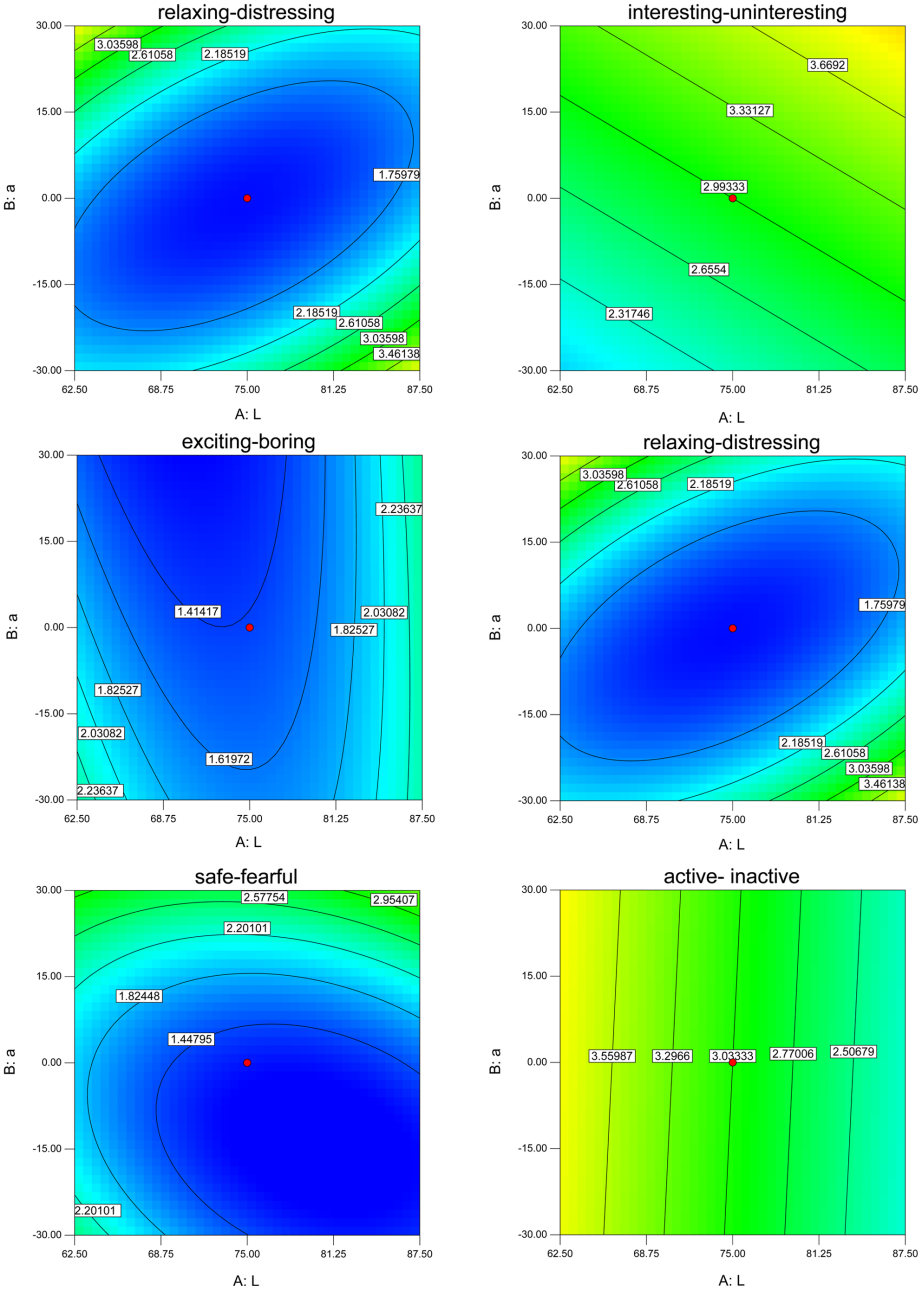
The contour plots for 6 responses.



Beside the numerical analysis, Design Expert also generated a graphical presentation to investigate the fitted model. For example, the contour plot for 6 responses is shown in Figure 6. Similar analyses can be done of the other responses. The resulting equations of the fitted model are:

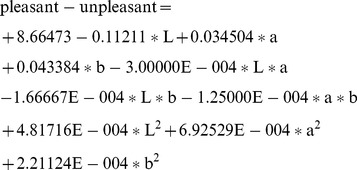
(1)

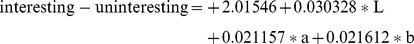
(2)

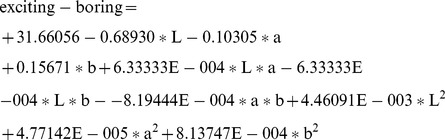
(3)

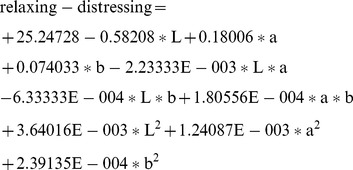
(4)

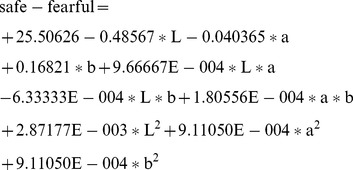
(5)

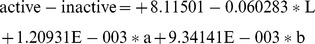
(6)


**Figure 7:**
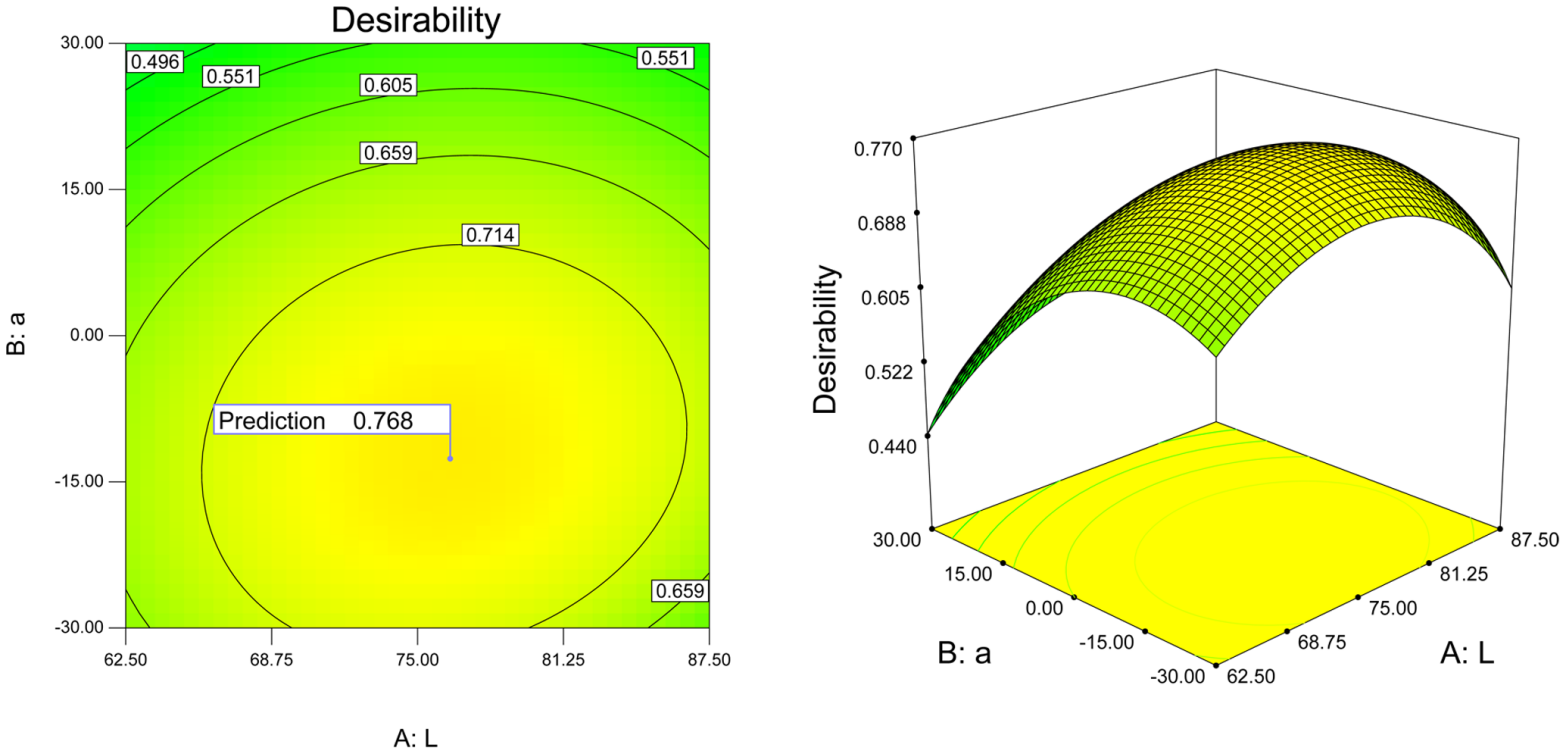
The contour and 3D plot of total desirability.

**Figure 8:**
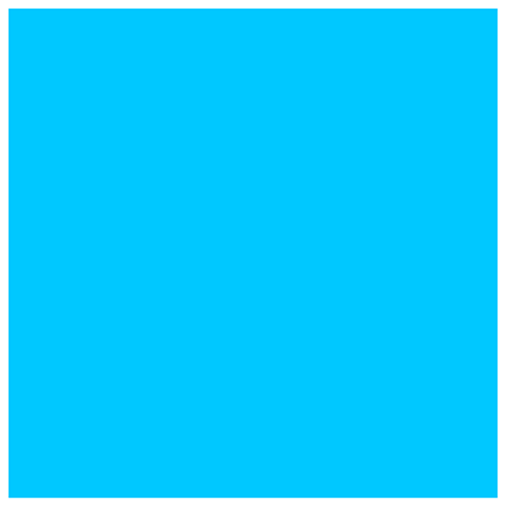
The optimal color solution.

Six responses are to be minimized. The contour plot of total desirability is shown in Figure 7. The flat plane in the three-dimensional plot indicates a region of optimum. Therefore, the optimal solution is chosen as L  =  76.40, a  =  – 12.65, b  =  – 77.00 (as shown in Figure 8 and Table 4).

**Table 4:** The optimal solution for color design.

*L*	*a*	*b*	Pleasant-unpleasant	Interesting-uninteresting	Exciting-boring	Relaxing-distressing	Safe-fearful	Active-inactive	Desirability
76.40	–12.65	–77.00	1.705	2.401	1.421	1.722	1.237	2.775	0.768

## Discussion

An important impetus for the growing international awareness of healthcare facility design has been mounting scientific evidence that certain environmental design strategies can promote improved outcomes.

Rubin and her colleagues [28] observed that enough quality research has appeared to justify the conclusion that there is suggestive evidence that aspects of the designed environment exert significant effects on clinical outcomes for patients. Johns Hopkins report indicated that most rigorous studies found positive links between environmental characteristics and patient outcomes [28].

The theoretical foundation of this research was based on the Conceptual Model of the Impact of Physical Environments on Patients’ Outcome proposed by Sherman, Shepley, and Varni [17]. This model indicates that physical environments can influence patients’ outcomes. They proposed that environmental satisfaction is a significant mediator between the built environment and patients’ outcome.

Experiments showed that blue and green are the preferred colors, and our results are also confirmed by other research. Hemphill [12] and Silver and Ferrante [29] reported blue to be the favorite color of both sexes and both age groups. In general, red walls have been associated with more dysphoria than blue-green walls [30]. Ward [31] reported that some colors, such as blue, decrease blood pressure, pulse and respiration, whereas others, such as red, tend to cause an increase in these biological functions. Research also supported that blue is the favorite color of both sexes of young and older adults, with green being preferred next by young adults.

There are some possible explanations for the results about the patients’ preference for partial black color. On the one hand, the patients saw black color as more pleasant and relaxing than white color, and perhaps the environment’s atmosphere may induce their intimate behavior. Furthermore, the relatively less pleasant atmosphere from white color may possibly inhibit their intimate behavior. On the other hand, the favorable impression of the psychologist might mediate the patients’ response. In the conditions with black color, the patients might disclose themselves more frequently, because they regarded the psychologist as more favorable than the conditions with white color.

In addition, psychologists should be aware of the physiological effects of color choices. For example, although cool colors may be desirable because they may decrease blood pressure and pulse rate, research indicates that the may also contribute to feelings of sadness and fatigue. Color theory suggests that psychologists should consider choosing wall/ceiling colors that are visually pleasing to them because many psychologists occupy the office on a daily basis. Balanced against the desire to please oneself is the desire to create a counseling space in which patients with different color preferences can all be comfortable.

In a recent review of research on color in environmental design, Pressly [32] concluded that there is a lack of experimental research approach on this topic. The methodology illustrated in this study is first step towards a better understanding of common guidelines optimizing color design in the applied field. Data from observational and critical incident approaches can generate viable hypothesis to be evaluated experimentally. Experiments have the flexibility to assess complex interactive, moderational, and meditational hypotheses, as well as more straightforward ones.

For example, experiments that use factorial designs can be very helpful in exploring whether the effect of one or more environmental components varies under differing levels of another component. We reviewed an investigation with such a design that found that the influence of counselor self-disclosure on perceptions of counselor credibility differed depending on whether there was a physical barrier between counselor and client [33]. Factorial experimental designs are useful in evaluating the validity of these and other complex, interactive predictions.

To apply the findings to actual counseling room design, we have to consider some points.

First, the patients were selected randomly in psychological clinic of general hospital. Thus, it is difficult to judge whether the differences of color would have the similar effects to patients who have serious depression and high anxiety. Consequently, further studies and observations that focused on persons who really come to therapy or counseling should be needed to test the effects of color in counseling settings.

Second, this study focused on the physical variables that would have impression on patients. Therefore, our findings may not always bring about the same outcomes as this study in actual counseling in which the number of people and types of conversation are varied. In actual counseling, therapists and counselors conduct various treatments, such as group counseling and family therapy. It is implicated that the patterns of the effects might change with the types of communication. Thus, the application of color to various types of communication should be examined more precisely in additional research.

Finally, this study highlighted the effects of color in a counseling room. Besides this factor, there may also be more effective physical issues to conduct counseling for patients. Further explorations of physical variables may stimulate or inhibit development of other physical factors and relationships among them.

From the practical point of view, the knowledge about environmental factors in counseling such as effects of color helps psychologists to practice their therapy more effectively. Like some researchers [34]–[36], we consider counseling color design as areas of overlap between environmental and clinical psychology and recommend that clinical psychologists, psychiatrists, and their organizations should be more conscious of environmental factors in their counseling rooms. Although many universities’ curriculums for official licenses of psychological counselors require no courses of environmental psychology, many psychologists want to know how to design their counseling room to make them more effective. In the future, educational programs for counselors should include courses related to studies about therapeutic environments that have been explored in environmental psychology.

## Conclusions

This paper has carried out research to apply experimental design and optimization theory to develop a methodology for color design of counseling room. The results enable us to understand patients’ preference in the color design and to develop method based upon optimization theory for creating color design more scientifically and effectively.
